# “If you show them love from an early age, they become healthier.” How do Hispanics in northern Indiana think about infant mortality and perinatal health?

**DOI:** 10.3389/fgwh.2026.1736936

**Published:** 2026-02-04

**Authors:** Jean Marie Sims Place, Liliana Quintero, Kalyn Renbarger, Kalayz'ana Roach, Grace Tagler

**Affiliations:** 1 Department of Nutrition and Health Science, Ball State University, Muncie, IN, United States; 2 Northern Indiana Hispanic Health Coalition, Elkhart, IN, United States; 3 School of Nursing, Purdue University, West Lafayette, IN, United States

**Keywords:** antenatal (ANC), infant mortality, latino (Hispanic), prenatal, prenatal care

## Abstract

**Background:**

Infant mortality, the death of a baby before its first birthday, is a concern in the Hispanic population in Indiana who have higher infant mortality rates than at the national level. Infrastructure and policy issues, effects of systemic racism and bias, lack of knowledge about perinatal health, and insecurity of the Hispanic community are issues challenging Hispanic infant health in Indiana. The purpose of this study was to describe the beliefs that comprise the Hispanic community's understanding about infant mortality.

**Method:**

This study used a qualitative descriptive design and data was analyzed using a Constructed Grounded Theory approach. Ten participants were recruited for an in-depth interview through a Hispanic-serving community coalition in northern Indiana. The interviews aimed to understand participants’ understanding of what constitutes a healthy pregnancy and a healthy infancy, as well as their understanding of the causes of infant mortality and perceptions of whether it can be prevented and by whom.

**Results:**

Three themes emerged from the data and included (1) Parental neglect plays a role in infant mortality; (2) Responsibility in parenting is central to prevention of infant mortality; and (3) Readiness matters when becoming pregnant and keeping child healthy.

**Conclusion:**

The Hispanic population may possess a distinct cultural understanding of the causes of infant mortality that does not align with expert knowledge. Results suggest participants place greater emphasis on postpartum care in the prevention of infant mortality and may not fully grasp the importance of prenatal care on postpartum outcomes. Targeted, culturally-salient communication and educational interventions are needed on infant mortality among the Hispanic population to ensure they can readily recognize factors that contribute to infant mortality.

## Background

### Infant mortality in the United States

Infant mortality, defined as the death of a baby before its first birthday, is a growing concern nationwide. The infant mortality rate (IMR), is calculated as the number of infant deaths per 1,000 live births, serving as a key indicator of a region's overall health. In Indiana, 524 infants died before their first birthday in 2023 (1). The infant mortality rate in Indiana for 2023 was 6.6 (per 1,000 live births), which is higher than the national rate at 5.6 (1). The leading cause of infant mortality in Indiana in 2023 included perinatal risks (48.5%), congenital anomalies (19.7%), sudden unexpected infant death (18.2%), other (10.6%), and assaults/injuries (3.0%) (1). Indiana ranks 13th in the nation for rates of infant mortality (2). Prenatal care is essential part of achieving healthy birth outcomes and lack of early prenatal care or no prenatal care is a direct contributor to infant mortality (3). Inadequate prenatal care is linked to increased risk of prematurity, stillbirth, early neonatal death, late neonatal death, and infant death, particularly for women of color (4). Prematurity and low birth weight, often detected and addressed through prenatal care, are top contributors to mortality risk (5).

### Racial and ethnic disparities with infant mortality

Hispanic infants in Indiana experience significant health disparities in infant mortality compared to White infants. Infrastructure and policy issues, effects of systemic racism and bias, insecurity of the Hispanic community, dissatisfaction with maternal care delivery, issues in navigating maternal healthcare, and limitations to holistic models of care have been identified as challenges for Hispanics in the perinatal period who live in Indiana (6). Among Hispanics in Indiana, the infant mortality rate was 7.0 (per 1,000 live births), higher than both the state (6.6) and national rate (5.6) in 2023 (1). Out of 79,000 live births in Indiana in 2023, 12.3% were Hispanic. The Hispanic population had the highest number of mothers on Medicaid (67.2%) compared to non-Hispanic Black (66.2%), and White (30.9%). Hispanic mothers were the least likely to receive early prenatal care (44.4%) compared to non-Hispanic Black (42.9%), and White (23.4%). Hispanic infants were more likely to experience low birth weight (8.4%) compared to White infants (7.4), and preterm birth (10.1) compared to White infants (10.5) (1).

### Beliefs and knowledge related to perinatal health and infant mortality

Hispanic women's community-based beliefs may impact their ability to access prenatal care in a timely manner and practice evidence-based care for their infants. For instance, Hispanic women and other women of color have reported preferring advice and support from their family members, partners and peers during their pregnancy, delivery, and postpartum over that from health care providers (7). While communities can provide emotional and tangible support for Hispanic women, gaps may exist in providing evidence based practices on how to prevent infant mortality. The risk for infant mortality can be reduced, but in some communities of color, the issue may not be perceived as preventable, with women rather seeing infant mortality through a fatalistic mindset (8). These beliefs may serve as a barrier to implementing effective strategies (e.g., safe sleep, breastfeeding) to reduce infant mortality for Hispanic women and other women of color (8).

Given the high rates of infant mortality in the Hispanic community in Indiana, health care providers who can effectively communicate about infant mortality and have an understanding of Hispanic community's beliefs is important. However, women have reported a lack of motivation to obtain care and poor knowledge of the impact of prenatal and preconception care on the health of the mother and offspring (9). Hispanic women may be reluctant to seek advice and care from healthcare providers due to negative perinatal experiences. For example, in a study of Hispanic women and other women of color, participants described a lack of knowledge surrounding pregnancy in addition to challenges communicating with clinicians in the perinatal period. Participants described experiences of being ignored, lack of empathy from clinicians, and the need for self-advocacy (7). Better understanding how the Hispanic community currently conceptualizes infant mortality and its causes equips practitioners to address gaps in knowledge or understanding.

### Purpose

The purpose of this study was to describe the beliefs that comprise the Hispanic community's understanding of infant mortality.

## Methods

### Design

This study used a qualitative design and data was analyzed using a Constructed Grounded Theory approach (10). This study was approved by the Ball State University institutional review board (IRB; 960087-2). Semi-structured, in-depth interviews are a typical method for data collection in qualitative studies (11). We selected Constructed Grounded Theory as an appropriate method to build a description of beliefs that comprise the Hispanic community's understanding of infant mortality. According to Charmaz (10), theories constructed from the data are interpretive portrayals of the studied phenomena built on research participants’ world views as well as the researcher assumptions that are brought to the research process and analysis. Our results are grounded in participants’ experiences, but also constructed from the perspective of the research team, including women who are not Hispanic, nor have given birth, as well as Hispanic women who have given birth. All members of the research team have college degrees. No one on the research team experienced the death of an infant before age one.

### Sample/setting

Ten participants were recruited from contacts of the Northern Indiana Hispanic Health Coalition (NIHHC), located in Elkhart, Indiana in the northern region of the state. Elkhart, Indiana has high infant mortality rates compared to the state averages. Among Hispanics, it has one of the highest infant mortality rates in Indiana at 6.9 (1). We recruited participants by reaching out by phone to past attendees of NIHHC-sponsored health fairs who fit our eligibility criteria (older adults, young mothers and fathers, and teenagers). We aimed to include a wide range of participant demographics, both in terms of age and parenting status, in an effort to capture a wide range of perspectives on a community's understanding of infant mortality. We employed maximum variation sampling to that end (12). Demographic information is included in Table 1.

**Table 1:** Demographic information for study participants (*n* = 9)^a^.

Sex	Number of participants
Female	6
Male	3
Age	
15–17	1
18–25	2
26–45	3
46–55	1
56+	2
Education level completed	
Elementary	2
Middle school	2
High school	3
College	1
Technical school	1
Country of origin	
USA	2
Mexico	6
Colombia	1
Have you ever been pregnant?	
Yes	5
No	1
Not applicable	3
How many children do you have?	
0	2
1	2
2	3
3	1
4	1

a Missing data for one participant.

### Procedures

If the contacted individuals agreed to participate, we asked the participants to arrive at the NIHHC at a designated day and time to sign an informed consent, complete a demographic form, and be interviewed. Participants received compensation in the form of a $20 gift card for completing the 30–40 min interview. The one-time, one-on-one, in-depth interviews were conducted by bilingual members of the research team (first and second authors) and audio-recorded with informed consent of the participants. Most interviews were conducted in Spanish, unless a participant preferred to speak in English. The 13-question interview guide was developed by the research team and aimed to assess participants’ understanding of what constitutes a healthy pregnancy and a healthy infancy, as well as their understanding of the causes of infant mortality and perceptions of whether it can be prevented and by whom (see Appendix for complete interview guide). While most questions remained the same across participants, some questions differed slightly depending on the sex and age of participants. Audio files were transcribed verbatim by members of the research team in the language the interview was conducted in and the text transcripts were thus analyzed in respective languages by members of the research team. For mono-lingual English speakers of the research team who helped code the transcripts, we used AI to translate the Spanish transcripts to English and then had a bi-lingual member of the research team conduct back-translation to ensure accuracy. English translations are the ones included in the results.

### Analysis

We used the constructed approach to Grounded Theory (10) to identify major themes in the data, with a goal of assessing the Hispanic community's understanding of infant mortality. Analysis was comprised of line-by-line codes for each of the 10 transcripts. Line-by-line coding is a way to carefully organize small segments of data from the transcripts into short summary phrases. We employed the constant comparative method, advanced by Glaser and Strauss (13), to compare data against data and establish distinctions between line-by-line codes. In the line-by-line coding process, we kept analytic memos that identified emerging ideas for the development of focused codes. Consistent with Charmaz's (10), these memos were useful to begin to synthesize and explain larger segments of data and to pull together ideas that coalesced within line-by-line codes. The resulting focused codes were then compared and contrasted through the constant comparative method, with attention to negative cases. We constantly checked the focused codes, to ensure they reflected the data, by returning to the transcripts to refine the codes within the data.

Data was saturated and no new themes were emerging from the data after the ten interviews were complete, thus we are confident we reached theoretical saturation with the results presented in the manuscript. As a way to establish the “confirmability” of the results, we used multiple coders from members of the research team (first, second, fourth, and fifth authors) to analyze the data. Focused codes between the coders were compared. The codes largely matched and were thus synthesized in the results.

## Results

Ten participants completed interviews. Demographic characteristics are displayed in Table 1. Three themes emerged from the data and included (1) Parental neglect plays a role in infant mortality; (2) Responsibility in parenting is central to prevention of infant mortality; and (3) Readiness matters when becoming pregnant and keeping children healthy.

### Theme 1: Parental neglect plays a role in infant mortality

Analysis from the in-depth interviews showed a conceptual connection between infant mortality and parental neglect. In other words, participants often linked the words “infant mortality” to the concept of negligence. Participants commented about the role of parental neglect in infant mortality, often blaming parents for the failure to care for their children adequately. One participant illustrated the connection by describing specific situations where lack of attention leads to a fatal accident, reinforcing the idea that infant mortality is a result of parental neglect:

There is the negligence of the parents that sometimes it is easy for us when they are, for example, one or two years old to leave them alone in a bathtub with water; in the bathtub they leave them bathing; there have been many cases; or as simple as the fact of the window cords, all of that, that is part of all that, that babies die because of that… (Participant “J”).

Another participant stated, “Infant mortality [is] when they die due to negligence. Because most of the time it is either the negligence of the parents or the negligence of the doctor, but it is negligence on either side” (Participant “LG”). This quote alludes to medical negligence, however, medical negligence was not salient enough as a subtheme, as evidenced in multiple transcripts, to be named as such in this study. Another participant (“Participant “L”) stated that infant mortality “could be parents or the mom is doing drugs during the pregnancy or just parents just not taking care of the baby like they should, having responsibilities, how do I say it, just not being responsible.” One participant summarized by saying, “As a bad parent, you don't pay attention to them [the infant] … and it is like it happens to worsen the little problem they had” (Participant “J”). A recurrent theme in the interviews was that parental neglect could contribute to infant mortality, with participants suggesting that the lack of proper care could be deadly.

### Theme 2: Responsibility in parenting is central to prevention of infant mortality

Due to an underlying belief that infant mortality has something to do with parental neglect, participants focused on the role of responsibility in parenting. There was a perception that parental responsibility is central to prevention of infant mortality. Participants suggested that keeping children away from external threats, such as open stairwells, and acting quickly if a medical illness or emergency arises, could prevent infant mortality. Prevention of infant mortality was linked to concepts such as responsibility, readiness to be a parent, and attentiveness. One participant stated:

Babies die when you leave them in the car seat in the hot in the summer… [You might say to yourself] ‘Ah! just two minutes!’ but you find yourself inside and there is a long line and you don't want to leave your things … and when five minutes go by, the car is too hot and the baby is already choking, intoxicated. (Participant “J”)

Another participant stated, “I think [infant mortality] happens because you don't take good care of your children because sometimes many people don't take the responsibility of having a child and we take it as a second thing, as a second priority” (Participant “F”). The participant linked unsafe sleep, poor nutrition, or failure to attend to concerns in pregnancy as a failure to be responsible.

### Theme 3: Readiness matters when becoming pregnant and keeping child healthy

Participants discussed the importance of being a parent when your body is physically capable to carry a child and when you are emotionally ready to be responsible and attentive to the child, such as having a willingness to take the child to the doctor's office and do other necessary things. Participant “L” stated, “I feel like it's not being ready for a baby, [that's] a big part of infant mortality.” Participant “A” discussed the components that need to be in place prior to having a child: “Besides emotional stability… social stability, economic stability, that is to say, [there needs to be] stability at all levels. Social, personal, financial, in every sense, because any of those things being wrong makes the pregnancy not as healthy as we would expect it to be.” Another participant summarized that a person needs to be “focused on raising their baby” (Participant “A”). In the case of being “too young” or unprepared, one participant discussed the sequelae: “So they are hit with all of these responsibilities they weren't ready for, and they get stressed out and do not take care of the baby like they should, and unfortunately babies could get sick or something bad happens to them, they fall or something like that” (Participant “F”).

## Discussion

The results of this study add to the limited body of research that exists to describe Hispanic community's beliefs about infant mortality. Some studies have revealed how community- based beliefs may impact Hispanic community member's decisions to implement evidence based practices related to infant mortality. For example, Hispanic women reported bedsharing as the result of generational advice passed onto them (14). Hispanic women have also acknowledged that familial perspectives and experiences were influential in shaping their decision to breastfeed their infant (15). However, studies are lacking that describe Hispanic community member's beliefs directly related to infant mortality. This may be related to discomfort when discussing the topic of infant mortality. In another study with African American community members discussing infant mortality, individuals tended to shy away from discussing the topic and not wanting to face the reality that infant mortality is a serious concern (8). This current study provided narratives of Hispanic community's beliefs about infant mortality that can be used to inform how health care providers educate both men and women on perinatal health.

While attentiveness, responsibility, and readiness were salient themes in caring for an infant, there was a failure to strongly connect prenatal health care with postpartum outcomes. Access to first-trimester prenatal care is a crucial step to ensuring healthy birth outcomes. Among the Hispanic population, early prenatal care can be faciliated by social support, adquate interpretation services, and effective health communication and education (16). The majority of participants did not mention indicators like full-term birth and birth weight as important to healthy pregnancies, even though they are the top contributors to infant mortality among the Hispanic/Latino(a) population. Risks that occur during pregnancy comprise the greatest cause of infant mortality among Hispanics in Indiana, making up 4.8 of 7.9 IMR (1). Based on the results, participants’ understanding of how pregnancy risks and prenatal care may contribute to outcomes is unclear or unexpressed. More than 60% (62%) of infant deaths in Indiana occurred during the neonatal period (0–27 days), with 71.1% of those deaths attributable to risks occuring during pregnancy (1). Among participants, there was a failure to understand these significant causes.

Participants instead focused on factors they could largely control in the postpartum period, such as ensuring safety in the infant's environment and avoiding dangers, although unsafe sleep environments was not included in their discussion despite sudden, unexplained infant death (SUIDs) accounting for a significant portion of the Hispanic IMR in Indiana (1). Participants tended to focus on injuries as a top cause of infant mortality, despite assaults and injuries being responsible for only 0.6% of deaths in the neonatal period and 8.2% in the post-neonatal period (1). Hispanics may know less about SUIDS than other racial/ethnic groups (17), thus more targeted information on safe sleep may be warranted and culturally and linguistically appropriate education could be disseminated via safe sleep community baby showers (18).

Adolescent pregnancy was mentioned indirectly as a concern in infant mortality, primarily because “being too young” was perceived to contribute to irresponsibility and poor decision making in childrearing. Stigmatizing attitudes towards teenage mothers is reflected in the literature (19). While we can work to reduce teenage pregnancies, it is important to also work to reduce stigma against teen mothers, which can cause parenting stress (20). Based on aggregate rates from 2013 through 2022, Indiana's age-specific birth rates for teenagers aged 15–19 was 15.7, compared to the national rate of 13.1 (1). Among Hispanics in Indiana, almost 7.8% of births in 2023 were to teenage moms (1). In Elkhart County, the rate for the same period was 26.6; this high rate explains that participants indeed recognized adolescent births as an important indicator in infant mortality. Infants are more likely to die when born to a teen mother, compared to being born to a woman over age 20. The reasons for deaths are related to preterm birth and low birthweight (21). While Hispanic youth are more likely to become pregnant if they express any degree of “wanting” a pregnancy (odds ratio 2.6), circumstances also play a role, such as the inability to access effective contraceptive use. Efforts to decrease pregnancy intention and increase access to contraception among teenagers should also be pursued (22).

There is room to broaden the conversation around infant mortality so the Hispanic population more readily recognizes the role of obesity, lack or limited prenatal care, and unsafe sleep practices as top factors contributing to infant mortality. For example, only about half (59.1%) of Hispanics in Indiana are receiving prenatal care in the first trimester, despite the importance of early prenatal care in prevention of low birthweight and preterm delivery (1). Structural barriers, such as lack of insurance or legal documentation, particularly for teen mothers, may prevent access to prenatal care (16). Unauthorized immigrants represented 4.1% of the total U.S. population and 27% of the foreign-born population with the countries of orgin Mexico, Guatemala, El Salvador, and Honduras having the largest populations in the US in 2023 (23). Mothers with unauthorized status experience variable access to pregnancy care, fear and stress regarding their status during pregnancy, and worse outcomes compared with other groups which include documented immigrants (24). In our study, instead of focusing on pregnancy risk, participants tended to focus on factors they perceived to be more within their control in the postpartum period.

### Limitations

The findings of this study should be considered within the context of its limitations. The study utilized a qualitative descriptive design with a small, localized sample of Hispanic participants in Indiana. Recruitment bias may be present due to participants being past-participants of the NIHHC health fairs, thus they may be more inclined to consume health-related information compared to the general public. This limits the extent to which the findings can be applied to other populations or settings beyond the study's local context. Second, since the interviews were conducted in both English and Spanish, there is a possibility of meaning being altered during translation or transcription. Although efforts were made to preserve participants’ original intent, subtle linguistic and cultural expressions may have been lost or interpreted differently. Additionally, self-report data is subject to social desirability bias. Participants may have framed their responses in ways they believed were expected or socially acceptable, particularly regarding sensitive topics like parental negligence or teen pregnancy and dpending on their comfort level speaking to a Hispanic or non-Hispanic interviewer and their ease in speaking the language they chose for the interview. Finally, we recognize the research team was the instrument through which the data was transmitted and interpreted and this comes with limitations based on the analytic experience of the researchers; in other words, we may not have been able to adequately capture participant meaning. The study offers valuable insight into culturally rooted beliefs and perceptions within a historically underrepresented group. By design, there was a large range of participant demographics included in the study (i.e., age, marital status, parental status). In general, there was broad agreement across the whole sample with regard to the the themes described in this study, however, potential subthemes salient to sub-populations in the sample should be explored in a future study.

## Conclusion

The findings of this study underscore the impact of perceptions; the Hispanic population may possess a distinct cultural understanding of the causes of infant mortality that does not align with expert knowledge; this may be influenced by their social positioning and migrant status in American society. Results suggest participants place greater emphasis on postpartum care in the prevention of infant mortality and may not fully grasp the importance of prenatal care on postpartum outcomes. Centering education for parents, coupled with adquate resources, is an essential part of the solution. Doing so through trusted messengers with knowledge of the local language and culture is vital. The evidence-based *Promotoras de Salud* model, similar to community health workers that uses trained peers to provide relevant, culturally tailored health education, has demonstrated success in behavior change, particularly in increasing folic acid and prenatal vitamin intake among Hispanic women (25). Culturally appropriate counseling and education prior to pregnancy and early in pregnancy is useful to communicate the importance of prenatal risk and protective factors (i.e., prenatal care, maternal nutrition, healthy weight, etc.). Group prenatal care is an evidence-based model to provide prenatal care and disseminate health information and has shown to increase perceptions of social support (26). Counseling and education should take into account maternal health literacy; higher health literacy is connected to improved self-efficacy (27) and thus communication should carefully consider language preference and use evidence-based, patient-centered strategies such as teach-back and teach-to-goal methods (28). Digital communication (social media, texting, mobile apps) promoted by trusted organizations have been shown to be effective at encouraging healthy behaviors, especially when members of the target population (e.g., Promotoras) are involved in the design of the content (29). Additionally, it is important to leverage informal information networks for health educational efforts, recognizing the significant influence of family and friends in shaping beliefs (30). This might look like purposefully integrating family members into health journeys and equipping them with credible information so they can be well-informed advocates and messengers. Further, safe sleep education—sometimes offered through community baby showers (18)—and teen pregnancy prevention efforts that prioritize private, confidential, one-on-one interactions with healthcare providers (31) are important corollary messaging that should be emphasized among Hispanic populations.

## Data Availability

The raw data supporting the conclusions of this article will be made available by the authors, without undue reservation.

## Appendix

Thank you for your time today! We will be discussing your thoughts, opinions, and perceptions about infant mortality, *or the deaths of infants before the age of 1*, in our community.

What do you think about when we say the word “infant mortality”?
To start off, we want to get a feel for how much of a problem you believe infant mortality is in our community. Would you rank it as a high priority problem, mid-priority problem, or low priority problem?
  (a) Why did you rank it that way?
Infant mortality (or the number of babies who die before the age of one) is very high in the Latino community, particularly this zip code: XXXXX. Why do you think that is?
Can you describe to me what you see as the major causes of infant mortality are among the Latino/Hispanic population in our community?
What are things you feel like contribute to having a healthy pregnancy or healthy baby?
  (a) **Let participants list ideas** **make sure they understand that we are interested in their opinions and there's no “right” answer for the purpose of this interview.**
    i. Do you think age at pregnancy matters to having a healthy pregnancy or baby? Why or why not?
    ii. What role does nutrition play in having a healthy pregnancy or healthy baby?
    iii. Does being overweight affect a pregnancy? Why or why not?
    iv. Do you think prenatal care is important? Why or why not? When should women go to prenatal care?
    v. What affect does tobacco/smoking have on a pregnancy or a baby?
    vi. Do you know when a full-term birth is? Why is a full-term birth important? What happens if the baby is not full-term? [Full-term births (i.e., when a woman delivers at or after 39 weeks of pregnancy)/preterm births (i.e., when a woman delivers before 37 weeks of pregnancy)]
    vii. What role does relationship stress have on a healthy pregnancy or healthy baby? Why does it matter?
    viii. Does mood or depression play a role in healthy pregnancies or healthy babies? Why or why not?
    ix. Is it okay to sleep in the same bed as a baby? Why or why not? Should you use bumper pads and other things in cribs? Why or why not? Is it okay for babies going to sleep with a pacifier? Why or why not? Can babies go to sleep on their back? Why or why not?
    x. Do you think breastfeeding should be prioritized? Why or why not?
    xi. Do you have ideas for how to prevent accidents that could harm your baby? Why ideas do you have?
What are some challenges or difficulties you think you might experience in taking care of your baby or keeping him/her healthy and safe?
  (a) What kind of challenges have you experienced in the past when you had a new baby?
  (b) Do other people in your community have trouble taking care of their babies? In what ways?
  (c) What about other people in your family?
What are some strengths you think the Latino population has in taking care of babies?
What are some resources (e.g., in community, family, friends) that will help you be a good mom, dad, or caretaker? What are some of those resources?
  (a) Do you use those resources? If not used, what are the barriers?
  (b) Do any of those services need to be adapted or changed to better serve you?
In your opinion, does the Latino community have enough information on sex education? Why or why not?
  (a) In your opinion, does the Latino community have enough information on pre-conception health? Why or why not?
  (b) In your opinion, does the Latino community have enough information on prenatal care? Why or why not?
  (c) In your opinion, does the Latino community have enough information on postpartum mental health? Why or why not?
  (d) In your opinion, does the Latino community have enough information on well-child visits? Why or why not?
If you could wave a wand and “change” something for moms, dads, families, and babies in our community, what would that be?
What can community agencies (hospitals, clinics, birthing centers, doulas, midwives, schools, employers) do to prevent infant mortality?
  (a) What can they do?
  (b) What about other agencies or organizations?
Do you think infant mortality is preventable? Why or why not?
  (a) Do you think other people think it is preventable? Why or why not?
What are some questions that moms, dads, or caregivers might have about taking care of their child during pregnancy or after that they might not feel comfortable asking?
Is there anything else that you think is important for other parents or other caregivers to consider about preventing infant mortality?
